# Electrode Materials, Structural Design, and Storage Mechanisms in Hybrid Supercapacitors

**DOI:** 10.3390/molecules28176432

**Published:** 2023-09-04

**Authors:** Xiaobing Du, Zhuanglong Lin, Xiaoxia Wang, Kaiyou Zhang, Hao Hu, Shuge Dai

**Affiliations:** 1 School of Physical and Engineering, Zhengzhou University, Zhengzhou 450052, China; 2 Guangxi Key Laboratory of Optical and Electronic Materials and Devices, College of Materials Science and Engineering, Guilin University of Technology, Guilin 541004, China; 3 School of Material Science and Engineering, Henan University of Science and Technology, Luoyang 471023, China

**Keywords:** hybrid supercapacitors, electrode materials, design structure, energy storage mechanism

## Abstract

Currently, energy storage systems are of great importance in daily life due to our dependence on portable electronic devices and hybrid electric vehicles. Among these energy storage systems, hybrid supercapacitor devices, constructed from a battery-type positive electrode and a capacitor-type negative electrode, have attracted widespread interest due to their potential applications. In general, they have a high energy density, a long cycling life, high safety, and environmental friendliness. This review first addresses the recent developments in state-of-the-art electrode materials, the structural design of electrodes, and the optimization of electrode performance. Then we summarize the possible classification of hybrid supercapacitor devices, and their potential applications. Finally, the fundamental theoretical aspects, charge-storage mechanism, and future developing trends are discussed. This review is intended to provide future research directions for the next generation of high-performance energy storage devices.

## 1. Introduction

In recent years, the increasing environmental problems and energy challenges have stimulated urgent demand for developing green, efficient, and sustainable energy sources, as well as revolutionary technologies associated with energy conversion and storage systems [1,2]. Among the diverse energy storage devices, supercapacitors (SCs) have received extensive attention due to their high power density, fast charge and discharge rates, and long-term cycling stability [3,4,5]. Generally, SCs can be classified as electrical double-layer capacitors (EDLCs), pseudocapacitors (PCs), or hybrid supercapacitors (HSCs) depending on the energy storage mechanism [6,7,8,9,10]. EDLCs collect energy through the ion absorption/desorption on the electrode/electrolyte interface without the charge transfer reaction [7,8]. PCs harvest energy through fast redox reactions at or near the surface of the electrode material [3,9]. Different charge storage mechanisms occur in the electrode materials of HSCs. For example, the negative electrode utilizes the double-layer storage mechanism (activated carbon, graphene), whereas the others accumulate charge by using fast redox reactions (typically transition metal oxides and hydroxides) [11,12,13,14]. HSCs have attracted enormous attention as they can provide excellent performance with higher energy and power densities at high charge/discharge rates [12,13]. More importantly, HSCs provide an important future opportunity for energy storage devices to meet the demands of both higher energy and power densities for powering portable electronic devices, hybrid electric vehicles, and industrial equipment.

At present, nanostructured transition metal oxides, sulfides, and hydroxides [15,16,17,18,19,20,21] are being widely explored as positive electrodes for HSCs. Such materials display a very fast charge/discharge rate to offer high power density. Unfortunately, many battery-type electrodes, such as Ni(OH)_2_ [22,23] or other materials, that exhibit faradaic behavior (even those that are electrochemically irreversible) have been considered as pseudocapacitive materials in many reports, which confuses the readers [24,25,26]. As suggested by Gogosti et al. [10], it is inappropriate to describe nickel-based oxides, sulfides, and hydroxides as pseudocapacitive electrode materials in alkaline aqueous electrolytes because they undergo faradaic reactions, where their electrochemical signature is analogous to that of a “battery” material. Therefore, the concept of “capacitance” (F) cannot be applied to purely faradaic behavior, and “capacity” (C or mAh) is the most appropriate and meaningful metric to represent the performance of such materials [26]. In addition, some researchers may mistakenly consider the HSCs as asymmetric supercapacitors (ASCs) that are based on two different supercapacitor-type electrodes (i.e., capacitive electrodes and/or pseudocapacitive electrodes), which also aggravates the confusion for readers [27]. The definition of an ASC device is very broad since it refers to every combination of positive and negative electrodes with the same nature regardless of the difference between the two electrodes (weight, thickness, material, etc.) [7]. However, an HSC device should be used when pairing two electrodes with different charge storage behaviors, such as one capacitive and the other faradaic, and the performance of such a device is in between a supercapacitor and a battery [27]. Some researchers have presented a well-rounded view in recent literature [27,28,29].

Herein, we will classify HSCs into several types based on the design and structure of the devices. It is well known that the performance of an energy storage device is determined mainly by the electrode materials. The design and development of nanomaterials and hybrid nanomaterials/nanostructures are considered as effective strategies to obtain advanced energy storage devices with high power, fast charging, and long cycle-life features [30,31]. More importantly, it enables us to develop a new generation of devices that approach the theoretical limit for electrochemical storage and deliver electrical energy rapidly and efficiently [30]. Although nanostructuring provides marvelous benefits, there are still some challenges in developing high-performance electrode nanomaterials for HSCs. For example, the electrode thickness of transition metal oxides and hydroxides (Ni(OH)_2_, NiS, NiO, etc.) is limited due to the low electrical and ionic conductivities of these materials, which retard the overall device kinetics. Both electronic and ionic conductivities are critical for increasing the rate performance of electrodes, especially when large and multivalent ions are used in electrolytes [32]. Therefore, it is necessary to improve electronic conduction by doping, partial reduction, and creating good electrical contact between nanomaterials and conductive additives [7]. Thus, some recent advances in electrode materials will be presented and discussed in this review article. Moreover, we will summarize the recent advances of HSCs, especially in the development of fundamental scientific principles and concepts. Then, we will provide a comprehensive summary of recent progress on electrode material design and burgeoning device constructions for high-performance HSCs. Finally, the future developing trends and perspectives, as well as the challenges, will also be discussed.

## 2. Recent Advances in Materials for Hybrid Supercapacitors

**Figure 1:**
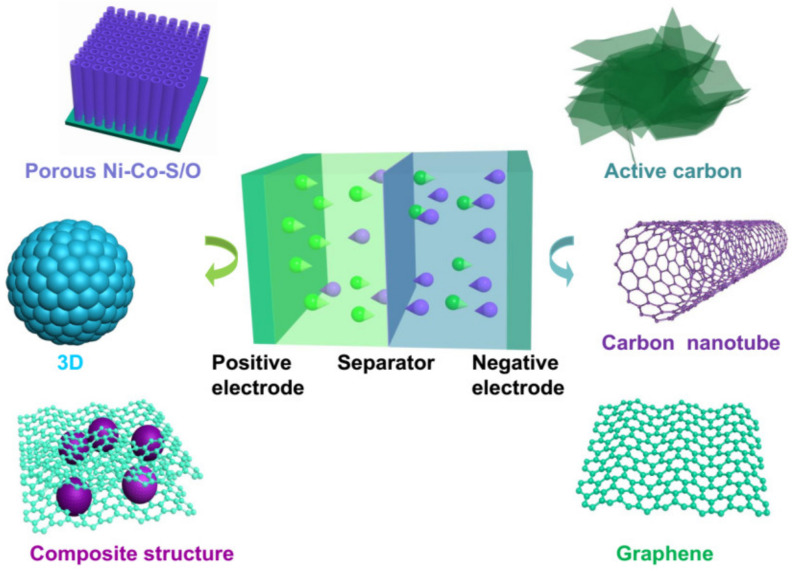
Illustration of a hybrid supercapacitor system.

HSCs are generally composed of three components (Figure 1): electrodes, electrolytes, and separators. The performance of HSCs is mainly determined by the electrochemical activity and kinetic features of the electrodes. To improve the energy and power density of HSCs, it is crucial to enhance the kinetics of ion and electron transport in electrodes and at the electrode/electrolyte interface [33]. Therefore, electrode materials, as the essential soul of the devices, play a decisive role in the performance of HSCs.

### 2.1. Positive Electrode Materials

The performance of a HSC device is mainly determined by the positive electrode materials [10]. In recent years, transition metal oxides/sulfides/hydroxides [34] have been considered as promising electrode materials for HSCs since they can provide a variety of oxidation states for fast surface redox reactions. 

#### 2.1.1. Nickel Oxides/Hydroxides/Sulfides

Recently, Ni-based oxides/hydroxides, such as NiO [35,36,37,38,39] and Ni(OH)_2_ [40,41,42,43,44], have been widely reported as electrode materials for HSCs due to their attractive theoretical specific capacity and potentially high-rate capability in alkaline aqueous solutions. NiO is a promising battery-type material due to its high theoretical specific capacity (1292 C g^−1^ in a potential window of 0.5 V), well-defined redox behavior, and low cost [38]. For instance, Ren et al. [45] prepared honeycomb-like mesoporous NiO microspheres and revealed a high specific capacitance of 635 C g^−1^ at 1 A g^−1^. Even at 5 A g^−1^, it also exhibited a high specific capacity of 472.5 C g^−1^ with 88.4% retention after 3500 cycles, demonstrating its superior performance. Cai et al. [46] prepared NiO nanoparticles and found a high specific capacity of 693 C g^−1^ at 1 A g^−1^, but the rate of capability could only retain 62% (430 C g^−1^) as the current density increased to 50 A g^−1^. The poor rate performance is caused by its low electrical conductivity. Although many recent efforts have been carried out on NiO electrodes, the acquired specific capacity is usually lower than the theoretical capacity of NiO. The relatively poor conductivity of NiO limited its specific capacity, and hindered the fast electron transport required for high charge–discharge rates.

**Figure 2:**
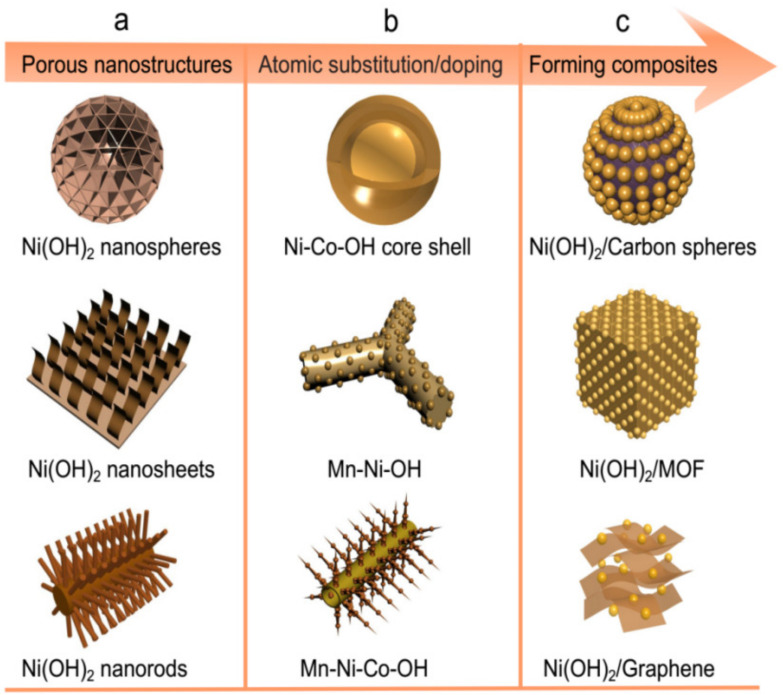
Illustration of nanoscale or porous structures of Ni(OH)_2_ (**a**), atomic substitution or doping (**b**), and fabricating a composite with carbon-based or other materials (**c**).

Compared to NiO materials, Ni(OH)_2_ has been considered as a promising candidate for HSCs due to its high theoretical capacity (1041 C g^−1^ in a potential window of 0.5 V), excellent redox behavior, ease of synthesis, abundant sources, low cost, and environmental friendliness [47]. Currently, many advances have been widely reported, as summarized in Table 1. During the last decades, numerous efforts have been devoted to fabricating high-performance electrodes based on Ni(OH)_2_ materials for energy storage devices, but there are still some challenging issues. Owing to its low conductivity, the Faradic redox reactions can only take place on its surface, and most of the reported Ni(OH)_2_ materials are inaccessible to electrolyte ions and remain as dead volumes in HSCs [48,49]. In recent years, many strategies have been explored to address this issue, including the synthesis of nanoscale or porous structures (Figure 2a), atomic substitution or doping (Figure 2b), and forming a composite with carbon-based or other materials (Figure 2c) [50,51]. For instance, the as-prepared hybrid electrode (Ni(OH)_2_/carbonnantube/polymer) by Jiang et al. [49] delivered an ultrahigh specific capacity of 1631 C g^−1^ at 5 mV s^−1^, excellent rate capability (71.9% capacity retention at 100 mV s^−1^), and long cycle life (85% capacitance retention after 20,000 cycles). In the hybrid, the conducting polymer coating contributes to stabilizing the whole electrode by reducing the dissolution of active materials, thus greatly improving the rate capability and cycling stability of the electrode. Fabricating a composite by incorporating highly conductive graphene nanosheets into Ni(OH)_2_ materials is considered as the most effective strategy to enhance the intrinsic properties of Ni(OH)_2_. Li et al. [52] reported a novel Ni(OH)_2_/rGO hybrid material, which not only exhibited a high specific capacity (1007.5 C g^−1^ at 0.5 A g^−1^), but also showed good life cycle stability (108% capacitance retention after 8000 cycles), revealing its good performance by incorporating rGO. Guo et al. [53] prepared a Ni(OH)_2_/rGO hybrid electrode and found a high specific capacity (1388 C g^−1^ at 2 A g^−1^) and remarkable rate capability (785 C g^−1^ at 50 A g^−1^). A Ni(OH)_2_-porous nitrogen-doped graphene hybrid architecture was also synthesized by Aghazadeh et al. [54]. The composite exhibited a specific capacity of 701 C g^−1^ and a capacity retention of 92.8% after 7000 cycles at 10 A g^−1^. In addition, the electrochemical performances of Ni(OH)_2_/rGO composites that have been reported thus far are compared in Table 2. It clearly reveals that, despite great achievement by hybridizing with rGO, Ni(OH)_2_ still requires further improvements, particularly in high-rate performance as well as in long cycle life.

Compared with corresponding oxides/hydroxides, transition metal sulfides have higher conductivity, mechanical and thermal stability, and richer redox reactions [89]. Over the past few years, transition metal sulfides (NiS, Ni_3_S_2_, MoS_2_, CoS, CuS, and FeS_2_, etc.) with superior optical, electrical, magnetic, and catalytic properties have been extensively used in the field of lithium ion batteries, SCs, and hydrogen evolution reaction catalysts [90,91,92,93]. Among numerous transition metal sulfides, nickel sulfides have been extensively investigated as positive electrode materials for HSCs due to their high electronic conductivity, low cost, and environmental sustainability [94,95,96,97,98]. For example, Zhang et al. [89] synthesized V-doped NiS_2_ with a high specific capacity of 981 C g^−1^ at 1 A g^−1^, and a good electrochemical cycling stability (100% of the capacity is retained after 6000 cycles). The as-prepared nanocrystalline β-NiS by Kushwaha et.al [95] exhibited a high specific capacity of 710 C g^−1^ at 1 A g^−1^, and long cycle stability (86% of the capacity retention after 10,000 cycles). Although nickel sulfides have been reported as promising positive electrodes for HSCs, they still suffer from some drawbacks, such as poor kinetics, polarization, dissolution of polysulfides in the electrolyte, thus reducing its conductivity, electrode pulverization, and the capacity loss [96,97]. In order to overcome these shortcomings, many researchers have devoted themselves to exploring the composites of nickel sulfide with carbonaceous materials. For instance, the as-prepared graphene-wrapped NiS nanocomposite by Zhang et al. [98] showed high specific capacity (1078.9 C g^−1^ at 2 A g^−1^) and good rate capacity (580.3 C g^−1^ at 15 A g^−1^), revealing that graphene plays a critical role in improving the performance of nickel sulfide at high current densities. In our recent work [12], we also found that the incorporation of graphene with nickel sulfide could stabilize its electrochemical properties. When integrated with rGO, the Ni_x_S_y_/rGO (NiS-Ni_3_S_2_-Ni_3_S_4_/rGO) composite electrode demonstrated not only higher specific capacity (807 C g^−1^ at 1 A g^−1^) but also better rate capability (~72% capacity retention as the current density was increased from 1 to 20 A g^−1^) [12]. Therefore, fabricating a composite with rGO is an effective strategy to enhance the specific capacity, rate capability, and cycling life of the whole electrode.

#### 2.1.2. Cobalt Oxides/Hydroxides

Among various transition metal oxides, Co_3_O_4_ has attracted wide attention for its high energy storage capacity (3560 F g^−1^), low cost, environmental friendliness, multiple valence sites, and high activity in water oxidation [99,100]. Electron and ion transport efficiency for charge storage in Co_3_O_4_-based pseudocapacitors mainly depends on electrode properties such as surface area, morphology, and alignment of nanocrystalline phases [101,102]. In the past decade, numerous Co_3_O_4_ nanostructures have been fabricated and tested for superior performance in the field of energy storage [103,104,105,106,107,108,109,110]. For example, Yang et al. [109] synthesized Pr-doped Co_3_O_4_ nanoflakes, which exhibited a high specific capacity of 640 C g^−1^ at a current density of 2 A g^−1^, and 64% of the capacity retention at a high current density of 10 A g^−1^. Zhang et al. [110] fabricated the Cl-doped Co_3_O_4_ hierarchical nanospheres and observed significant performance with an ultrahigh specific capacity of 814 C g^−1^ at a current density of 1 A g^−1^, high-rate capability (63.2% capacity retention at 32 A g^−1^) and good cycling stability. However, the observed specific capacity values for Co_3_O_4_ are much lower than their theoretical values, and the specific capacity usually severely decays at high charge/discharge currents. Therefore, it is an ongoing challenge to further improve its specific capacity and rate capability.

Cobalt hydroxide (Co(OH)_2_) is another kind of cobalt compound that has been widely investigated for its rich redox reactions [111]. Compared with nickel oxide and hydroxide, cobalt hydroxide provides more electrons when a redox reaction is going on [112,113,114]. Furthermore, hydrotalcite-like cobalt hydroxide usually shows a positively charged Co(OH)_2−x_ layer and an interlayered gallery with negatively charged anions (e.g., Cl^−^, SO4^2−^, NO^3−^) [115,116,117,118]. Recently, Xu et al. [117] reported the preparation of α-Co(OH)_2_ nanoparticles by cobalt zeolitic-imidazolate frameworks (ZIF-67) hydrolyzation, and the as-prepared α-Co(OH)_2_ nanoparticles presented superior specific capacity of 314 C g^−1^ at a low current density of 1 A g^−1^, high rate capability (77% of capacity retention at 20 A g^−1^) and good cycling stability (100% of capacity retention after 20,000 cycles). In order to further improve the cycling stability, some researchers have tried to fabricate the hybrids. For instance, Gan et al. [119] recently reported the Co(OH)_2_/CoS hybrid nanostructure, which displays high rate capability (75.8% capacity retention as the current density was increased from 0.5 to 10 A g^−1^) and long cycling stability. Wang et al. [120] synthesized a Co(OH)_2_/nitrogen-doped porous graphene composite and realized ultrahigh specific capacity (1144 C g^−1^ at 2 A g^−1^), and long cycling stability (95.9% of capacity retention after 4000 cycles).

#### 2.1.3. Multi-Metal Compounds

Owing to the multiple oxidation states and the synergistic effects between various metal ions, the multi-metal compounds show superior electrochemical performance for energy storage [12]. Generally, multi-metal compounds can be divided into multi-metal oxides, sulfides, and hydroxides. Multi-metal oxides, such as NiCo_2_O_4_ [121,122,123], ZnCo_2_O_4_ [124,125,126,127], MnMoO_4_ [128,129,130], and CoMoO_4_ [131,132,133,134], have been widely explored for energy conversion and storage. Among these multi-metal oxides, the NiCo_2_O_4_ nanomaterials have attracted increasing attention due to their merits of higher electrical conductivity, and higher electrochemical activity, which would offer richer redox reactions, including contributions from both Ni^2+^/Ni^3+^ and Co^3+^/Co^4+^ redox couples in the materials [121,122,123]. For example, Shen et al. [135] that reported the highly uniform NiCo_2_O_4_ hollow spheres exhibited a high specific capacity of 541 C g^−1^ at 1 A g^−1^, and excellent rate performance (74.8% of capacity retention from 1 A g^−1^ to 15 A g^−1^). In addition, it also demonstrated good cycling stability with 94.7% of capacity retention after 4000 cycles of continuous charge-discharge testing at the continuous charge-discharge testing at a current density of 5 A g^−1^. All these superior performances are caused by the advantageous structural features of these NiCo_2_O_4_ hollow spheres [135]. Ji et al. [136] reported a NiCo_2_O_4_ positive electrode material with an urchin-like hollow hierarchical microsphere structure, which delivered a high capacity of 424 C g^−1^ at 0.5 A g^−1^ and satisfactory rate capability (62.6% capacity retention from 0.5 A g^−1^ to 10 A g^−1^). To further enhance the electrochemical performance of NiCo_2_O_4_, many researchers have tried to design 3D porous hybrid electrode architectures by incorporating carbon materials. This hybrid architecture could solve the intrinsic poor conductivity and inevitable agglomeration of NiCo_2_O_4_ electrode materials [137]. For example, Li et al. [137] prepared the layered NiCo_2_O_4_/RGO nanocomposite and achieved an ultrahigh specific capacity of 694 C g^−1^ at 0.5 A g^−1^ and excellent cycle life with 90.2% capacity retention after 20,000 cycles at 5 A g^−1^. Al-Rubaye et al. [138] recently reported the NiCo_2_O_4_-rGO nanocomposite, which consists of NiCo_2_O_4_ hexagons wrapped in conducting rGO sheets, which exhibited a high specific capacity of 533 C g^−1^ at 2 A g^−1^ and excellent cycling stability with 98% capacity retention after 10,000 cycles. Sun et al. [139] reported the NiCo_2_O_4_ nanoparticle/three-dimensional porous graphene (NiCo_2_O_4_/3D-G) composite by a facile hydrothermal method combined with subsequent annealing treatment. The obtained NiCo_2_O_4_/3D-G hybrid electrode displayed a high specific capacity of 920 C g^−1^ at 1 A g^−1^. When being used as a positive electrode for HSC, the NiCo_2_O_4_/3D-G//rGO HSC device exhibited a high energy density of 73.8 W h kg^−1^ at a power density of 800 W kg^−1^ and long cycle stability with 94.3% capacity retention after 5000 cycles [139].

**Figure 3:**
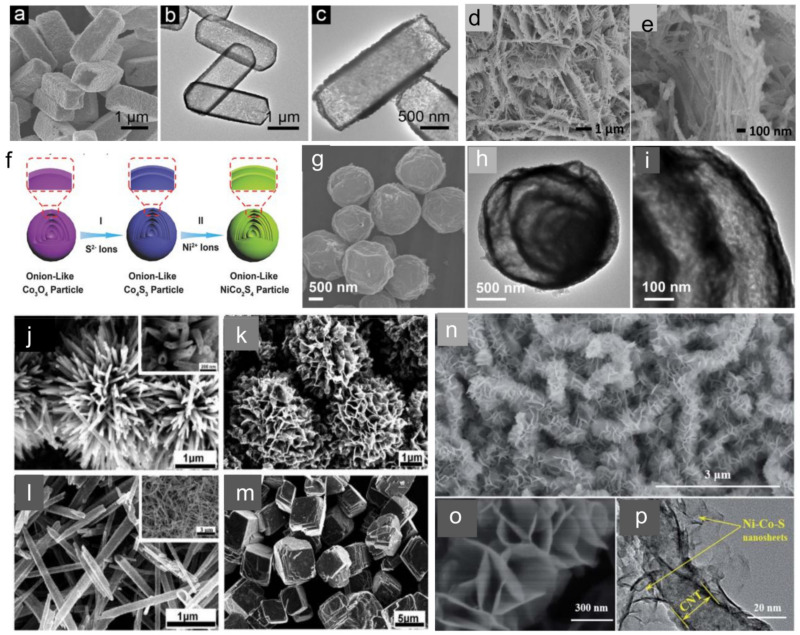
(**a**) SEM and (**b**,**c**) TEM images of NiCo_2_S_4_ hollow prisms [168] (Copyright 2014 WILEY-VCH Verlag GmbH & Co. KGaA, Weinheim). (**d**) SEM and (**e**) TEM images of NiCo_2_S_4_ nanosheet hetero-structured arrays [169] (Copyright 2015 Elsevier Ltd., all rights reserved). (**f**) Schematic illustration of the formation process; (**g**–**i**) TEM images of onion-like NiCo_2_S_4_ hollow microspheres [170] (Copyright 2017 WILEY-VCH Verlag GmbH & Co. KGaA, Weinheim). (**j**–**m**) SEM images of NiCo_2_S_4_ with different morphologies: urchin (**j**), flower (**k**), tube (**l**), and cubic (**m**) [146] (Copyright 2014, The Royal Society of Chemistry). (**n**,**o**) SEM images of the core/shell-structured CNTs@Ni-Co-S hybrids. (**p**) TEM image for detailed core/shell structure information of the CNTs@Ni-Co-S hybrids [144] (Copyright 2016: The Royal Society of Chemistry).

Compared to the multi-metal oxides, the multi-metal sulfides show better electrical conductivity, mechanical and thermal stability, and higher electrochemical activity [140,141,142]. Recently, many advances have been widely reported, including Ni-Co-S [143,144,145,146,147,148], KCu_7_S_4_ [149,150,151,152], CuCo_2_S_4_ [153,154,155], Zn-Co-S [156,157], Mn-Co-S [158,159,160,161], CuSbS_2_ [162,163], SnCoS_2_ [164,165,166]. Among these multi-metal sulfides, nickel–cobalt sulfides (NiCo_2_S_4_) have been reported with a much higher conductivity and richer redox reactions due to the electrochemical contributions from both nickel and cobalt ions, resulting in better electrochemical performance [167,168,169]. For example, Yu et al. [168] reported a facile self-sacrificial template method to synthesize uniform 3D NiCo_2_S_4_ hollow nanoprisms with controllable composition. SEM (Figure 3a) and TEM (Figure 3b,c) images display the rough surface and well-defined inner cavities of the NiCo_2_S_4_ hollow nanoprisms. When evaluated as an electrode for HSCs, the NiCo_2_S_4_ hollow nanoprisms show a high capacity (447.6 C g^−1^ at 1 A g^−1^) and remarkable rate capability (65.4% retention of initial capacity from 1 to 20 A g^−1^). Wang et al. [169] prepared NiCo_2_S_4_ nanosheets (Figure 3d,e) and realized an ultrahigh specific capacity of 1384 C g^−1^ at 2 mA cm^−2^. Guan et al. [170] reported an onion-like NiCo_2_S_4_ particle with unique hollow structured shells using onion-like metal oxide particles as the precursor, as illustrated in Figure 3f. As shown in the TEM images in Figure 3g–i, the onion-like NiCo_2_S_4_ particles are composed of several crumpled layers. Owing to its intriguing structural features, the obtained onion-like NiCo_2_S_4_ exhibited good electrochemical performance with a high specific capacity of 508 C g^−1^ and long cycling stability with 87% capacity retention after 10,000 cycles [170]. Typical CV curves of the NiCo_2_S_4_ electrode at different scanning rates are shown in Figure 3f. All these curves exhibit a typical battery-like feature. Figure 3g shows the galvanostatic charge-discharge curves of the electrode at various current densities in a potential window of 0–0.5 V. All these curves show a well-defined discharge voltage plateau at around 0.2–0.3 V, further demonstrating a good electrochemical battery-type characteristic and superior reversible redox reaction. Zhang et al. [146] reported a facile hydrothermal approach for the shape-controlled synthesis of NiCo_2_S_4_ architectures with four different morphologies of urchin (Figure 3j), tube (Figure 3k), flower (Figure 3l), and cubic-like (Figure 3m) microstructures. Among these architectures, the tube-like NiCo_2_S_4_ electrode exhibited the best specific capacity value of 419 C g^−1^ at a current density of 3A g^−1^ [146]. Peng et al. [144] reported a facile two-step method to synthesize 3D core/shell-structured composites (CNTs@Ni-Co-S). Figure 3n,o exhibits the SEM images of the Ni@CNTs@Ni-Co-S composite, revealing the skeleton of CNTs to form a clear and with each other to form a highly open structure, providing abundant accessibltypical core-shell hybrid structure. These nanosheets are interconnected pathways for electrolyte ions [144]. The TEM image (Figure 3p) presents the detailed information for the core/shell hierarchical nanostructures of CNTs@Ni-Co-S composites. At the interface between CNTs and Ni-Co-S nanosheets, Ni-Co-S nanosheets are found to adhere to the surface of CNTs robustly, which is favorable for electron transfer through CNTs to Ni-Co-S nanosheets [144]. The as-prepared composite electrode delivered a high specific capacity of 222 mA h g^−1^ at 4 A g^−1^ and excellent rate capability (193 mA h g^−1^ at 50 A g^−1^) [144].

**Figure 4:**
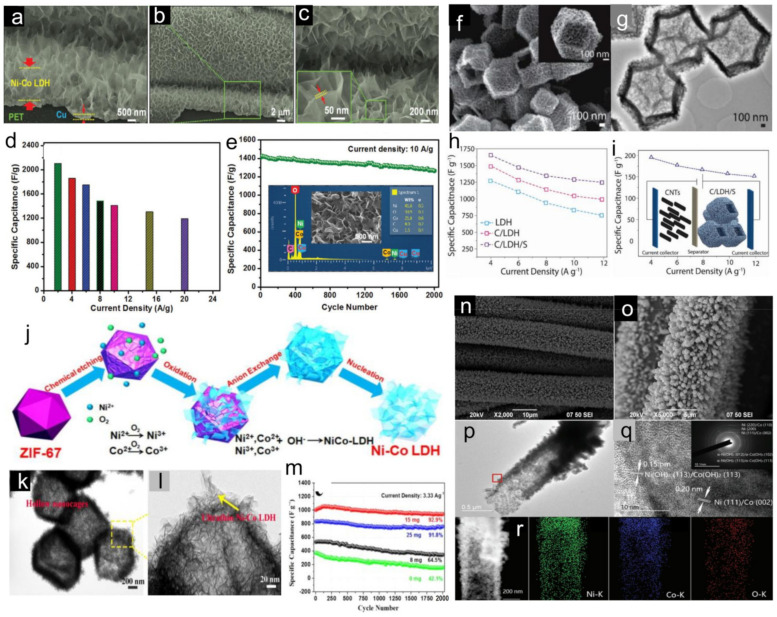
(**a**–**c**) FESEM images of the Ni-Co LDH NSs/CTs. (**d**) Specific capacitance values as a function of current density of Ni-Co LDH NSs/CTs. (**e**) Cycling performance of the sample at a current density of 10 A g^−1^ in 1 M KOH electrolyte solution. The inset of (**e**) shows the EDX spectrum and FE-SEM image of Ni-Co LDH NSs/CTs after the cycling process [173] (Copyright 2016: The Royal Society of Chemistry). SEM (**f**) and TEM (**g**) images of NiCo-LDH/Co_9_S_8_ polyhedrons. (**h**) Specific capacitances as a function of the current density. (**i**) Calculated specific capacitance values for NiCo-LDH/Co_9_S_8_//CNTs hybrid supercapacitor cells. Inset is a schematic illustration of the cells [174] (Copyright 2017 WILEY-VCH Verlag GmbH & Co. KGaA, Weinheim). (**j**) Schematic illustration of the possible mechanism of reaction involved in forming Ni-Co LDH. (**k**,**l**) TEM images of Ni-Co LDH hollow nanocages/graphene composites. (**m**) Cycling stability tests of the Ni-Co LDH/graphene composite with different graphene masses [175] (Copyright 2017 American Chemical Society). (**n**,**o**) SEM images of Ni-Co@Ni-Co LDH natube arraies. TEM (**p**) and HRTEM (**q**) images of Ni-Co@Ni-Co LDH NTAs. (**r**) TEM elemental mapping of Ni, Co, and O. (Ni:Co = 5:5) [176] (Copyright 2017 WILEY-VCH Verlag GmbH & Co. KGaA, Weinheim).

In recent years, multi-metal layered double hydroxides (LDH) have also attracted much more attention in the design of electrode materials for HSCs. Various successful achievements have been widely reported in the literature [171,172,173,174,175,176]. For example, Nagaraju et al. [173] reported a facile and cost-effective process to obtain well-assembled porous Ni-Co LDH nanosheets on conductive textile substrates (Ni-Co LDH NSs/CTs) using a two-electrode system-based electrochemical deposition method. SEM images indicate that the entire surface of the CTs has an average height of 1.2–1.3 μm (Figure 4a,b), and the surfaces of these nanosheets are smooth with a thickness of approximately 10–15 nm (Figure 4c) [173]. The as-prepared Ni-Co LDH NSs/CTs showed high specific capacity of (Figure 4d) and good cycling stability (Figure 4e). To further improve the performance, some researchers tried to incorporate foreign anions (e.g., nitrate, chloride, sulfate, acetate, etc.) into the interlayer region, or grow the active materials on nano-architectured carbon-based materials (graphene, carbon nanotube, etc.) to form hybrids [174,175,176]. Figure 4f,g show the polyhedron morphology from typical SEM and TEM images of the NiCo-LDH/Co_9_S_8_ sample, respectively. The as-prepared NiCo-LDH/Co_9_S_8_ hybrid system collectively presents an ideal porous structure, rich redox chemistry, and high electrical conductivity matrix, which deliver a high specific capacity of 743.8 C g^−1^ (1653 F g^−1^) at 4 A g^−1^ (Figure 4h) [174]. Moreover, a hybrid supercapacitor device based on NiCo-LDH/Co_9_S_8_ polyhedrons and carbon nanotubes delivers a high specific capacitance of 194 F g^−1^ and superior rate capability of 77.8% (Figure 4i) [174]. Bai et al. [175] reported the synthesis and characterization of Ni-Co LDH hollow nanocages, which are deposited on commercial graphene nanosheets derived from ZIF-67/graphene pristine material via a structure-induced anisotropic chemical etching at elevated temperature (Figure 4j). Figure 4k,l show the SEM images of the Ni-Co LDH/graphene composite, indicating that the shell of the hollow structure is composed of interconnected nanosheets with ultrathin thickness. The unique nanocomposite electrode delivered a high specific capacity of 759 C g^−1^ (1265 F g^−1^) at 1 A g^−1^ (Figure 4m). Recently, Liu et al. [176] synthesized the Ni-Co@Ni-Co LDH nanotube arrays (NTAs) by using ZnO nanorod arrays (ZnO NRAs) grown on CFC as a template. SEM images show that the Ni-Co@Ni-Co LDH nanorod is a fungus composed of a big top and a small body (Figure 4n,o). The TEM image (Figure 4p) indicates that the top of the Ni-Co@Ni-Co LDH nanotube has a diameter of ~700 nm and consists of many nanosheets, whereas the body of the nanotube has a diameter of ~550 nm and consists of nanocrystals (20~30 nm in size) [176]. The SAED pattern (inset of Figure 4q) reveals that the body of the Ni-Co@Ni-Co LDH NTA consists of many nanocrystals, and the EDX mapping (Figure 4r) shows that Ni, Co, and O are well-dispersed and homogeneously mixed in the NTA. Owing to their intriguing structural features, the Ni-Co@Ni-Co LDH NTAs deliver excellent rate performance (82.1% capacity retention from 1 to 20 A g^−1^) with an ultrahigh specific capacity of 1207 C g^−1^ at 1 A g^−1^ [176]. The Ni-Co@Ni-Co LDH NTAs are further paired with CNFs to fabricate HSCs, which delivered high specific capacitances of 280, 253, and 220 F g^−1^ at discharge current densities of 5, 10, and 20 A g^−1^, respectively. Most remarkably, the device exhibited an ultrahigh energy density of 100 Wh kg^−1^ at a power density of 1500 W kg^−1^ [176]. In addition to Ni and Co, some researchers also reported the effect of other transition metals (i.e., Zn, Mn, Al, and Fe) contents in Ni or Co electrode materials, as illustrated in Table 3.

### 2.2. Negative Electrode Materials

The negative electrode material is also crucial in developing high-performance HSCs with high energy density and excellent rate capability. Since the different mass ratios will affect the overall capacitance of the HSC device [202,203], to balance the charges stored on the two electrodes of HSCs, the matching ratio of positive and negative electrodes should be accurately calculated. Carbon materials, such as activated carbon (AC), carbon nanotubes (CNTs), and reduced graphene oxide (rGO), are widely utilized for electrode materials in SCs due to their easy accessibility, good processing ability, large surface area/porosity, low electrical resistivity, robust surface chemical environment, physicochemical stability, and low cost [33]. Currently, the most commonly used electro-active materials in HSC electrodes are AC, CNTs, and rGO materials.

#### 2.2.1. Activated Carbon Materials

AC is the most commonly used negative electrode material in HSCs because of its low cost and large surface area. At present, the AC electrodes have been applied to commercial SCs with high power density. Many recent advances in AC-based HSCs have been widely reported, as summarized in Table 4. The capacitance of AC is not linearly related to its surface area and pores sizes, such that the specific capacitance of micropores is larger than that of mesopores [33,203]. Therefore, controlling the pore size distribution of AC electrodes is very important. Kierzek et al. [204] prepared microporous AC with a surface area in the 1900–3200 m^2^ g^−1^ range and a pore volume of 1.05 to 1.61 cm^3^ g^−1^. The capacitance values ranging from 200 to 320 F g^−1^ were achieved compared with the 240 F g^−1^ of the commercially available ACs [204,205]. AC with remarkable performance, similar to SC electrodes, has also been prepared using other methods. For instance, Zhang et al. [206] prepared AC by the ZnCl_2_ activation method, and the material exhibited a high surface area of 1935 m^2^ g^−1^ and a total pore volume of 1.02 cm^3^ g^−1^. Moreover, it showed a high specific capacitance of 374 F g^−1^ (1 mol L^−1^ H_2_SO_4_ electrolyte), excellent capacity retention, and long cycling stability. In brief, although AC has a long history of usage and production, its structural and chemical characteristics are experiencing continual evolution to meet the requirements of more demanding emergent applications [205].

#### 2.2.2. Carbon Nanotube Materials

CNTs have been widely studied for SCs owing to their porous structure, high surface area, good electrical conductivity, and low density [221,222,223]. Owing to their unique tubular structures and the high density of mesopores, they exhibit much higher specific capacitance than ACs [224]. Compared to multiwalled CNTs, single-walled CNTs exhibit better electrochemical properties because of their large specific surface area (~1600 m^2^ g^−1^), high aspect ratio, fast charge transport, and large accessibility of electrolyte ions [225,226,227]. Recently, Wang et al. [227] reported hierarchically porous CNTs by a simple carbonization treatment, which displayed a high specific surface area of 1419 m^2^ g^−1^ and hierarchical micro-/meso-/macroporosity. This unique porous architecture delivered an ultrahigh specific capacitance of 286 F g^−1^ at 0.1 A g^−1^, and excellent rate capability (~71% capacity retention from 0.1 to 50 A g^−1^) [227]. To increase the energy and power density of devices, other strategies have also been -employed, such as atomic doping and combining CNTs with other materials (e.g., metal oxides, ACs, and graphehe) [228,229,230,231]. For example, Kim et al. [230] recently reported a polyimide/MWCNT composite electrode with a high specific capacitance of 333.4 F g^−1^ at 1 A g^−1^. Jin et al. [231] reported a polyaniline/carbon nanotubes/graphene/polyester hybrid electrode with a high areal capacitance of 791 mF cm^−2^ at a current density of 1.5 mA cm^−2^. Although various types of research have been carried out on CNTs for HSCs, most of the reported electrodes are often in powdered form or have a disordered texture with poor interconnectivity among micro-/mesoporous structures, which leads to a low specific capacitance and high internal resistance, thus resulting in a much lower energy and power density for devices [232,233,234]. Therefore, it is still a great challenge to further improve its performance.

#### 2.2.3. Reduced Graphene Oxide Materials and Their Hybrids

**Figure 5:**
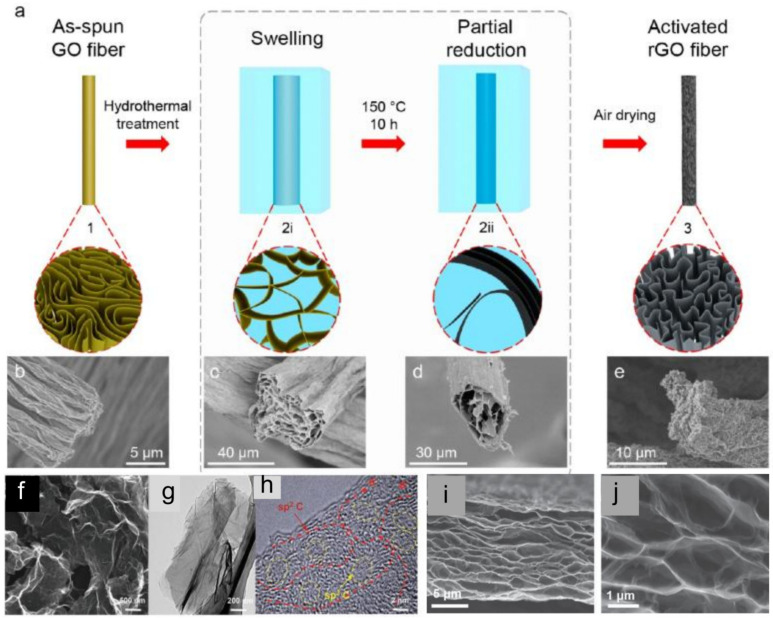
(**a**) Schematic diagram showing the structural evolution of graphene fibers during the hydrothermal activation process. Crosssectional SEM images of graphene fibers in the corresponding stages: (**b**) as-spun GO fiber, (**c**) swelled GO gel fiber at the beginning of hydrothermal treatment, (**d**) rGO gel fiber at the end of hydrothermal treatment, and (**e**) the resulted hierarchical rGO fiber after air-drying [249] (Copyright 2017 American Chemical Society). (**f**) SEM image of the GO-160-8D sample. (**g**) TEM image of the GO-160-8D sample. (**h**) high-magnification TEM image [250] (Copyright 2016 WILEY-VCH Verlag GmbH & Co. KGaA, Weinheim). (**i**,**j**) Specific capacitances of GO-160-8D under mass loadings of 2 and 10 mg cm^−2^, and that of commercial activated carbon (YP-50) under a mass loading of 10 mg cm^−2^ [256] (Copyright 2017 WILEY-VCH Verlag GmbH & Co. KGaA, Weinheim).

Another promising negative electrode material for HSCs is graphene. Graphene, a two-dimensional carbon sheet with monoatomic layer thickness, has been widely explored as an ideal electrode material for SCs due to its unique properties, including its high theoretical surface area (2630 m^2^ g^−1^) and high in-plane electrical conductivity [235,236]. It has brought a sensational revolution in the field of energy storage and conversion. To date, various routes have been developed to fabricate graphene sheets, such as blade-coating, spray-coating, layer-by-layer assembly, interfacial self-assembly, and filtration assembly [237,238,239,240,241,242]. In principle, a supercapacitor based on graphene is capable of achieving a theoretical electrochemical double layer capacitance as high as 550 F g^−1^ [243,244]. However, the practical performance of graphene is far below the ideal one due to various reasons. One of the main reasons is that the 2D layered graphene sheets can easily restack to form dense lamellar microstructures, which greatly decreases the specific surface area of the original graphene sheets, causes inferior ion transport capabilities, and renders a substantial number of active sites inaccessible to reactants [245,246,247,248]. Therefore, a number of strategies have been developed to prevent aggregation of graphene sheets so as to increase surface area and promote the transport of electrolyte ions, including fabricating 3D porous nanostructures [249,250], nitrogen doping [251,252,253], and surface modification using molecular modifiers [254,255]. For example, Li et al. [249] fabricated electrochemically active graphene fiber fabrics (GFFs) with a hierarchical morphology by using a hydrothermal activation strategy (Figure 5a–e). In such a process, crumpling of the graphene sheets within graphene fibers made for efficient activation on GFFs with a largely increased specific surface area [249]. Recently, Liu et al. [250] reported a novel strategy for the synthesis of pseudocapacitive oxygen clusters in graphene frameworks with “paddy land” structures through low-temperature thermal annealing of graphene oxide (Figure 5f–h). The SEM image (Figure 5i) clearly shows the typical flakelike morphology, with the lateral size ranging from 500 nm to a few micrometers. Moreover, the TEM image (Figure 5j) indicates the smooth surface of the GO-160-8D with layer stacking structure. Moreover, a high-magnification TEM image clearly demonstrates that sp^3^ carbon domains consisting of oxygen clusters are well distributed in the continuous sp^2^ carbon network. The as-prepared functionalized graphene shows ultrahigh specific capacitance of 436 F g^−1^ at 0.5 A g^−1^, excellent rate performance (261 F g^−1^ at 50 A g^−1^) and long cycling stability (94% of capacitance retention after 10,000 cycles) [250]. Shao et al. [256] reported a 3D porous rGO film with a high conductivity (1905 S m^−1^) and good tensile strength. The open surfaces of the 3D porous rGO films can be easily accessed by electrolyte ions without a diffusion limit, which guarantees a large capacitance at high current density/scan rate [256]. 

**Figure 6:**
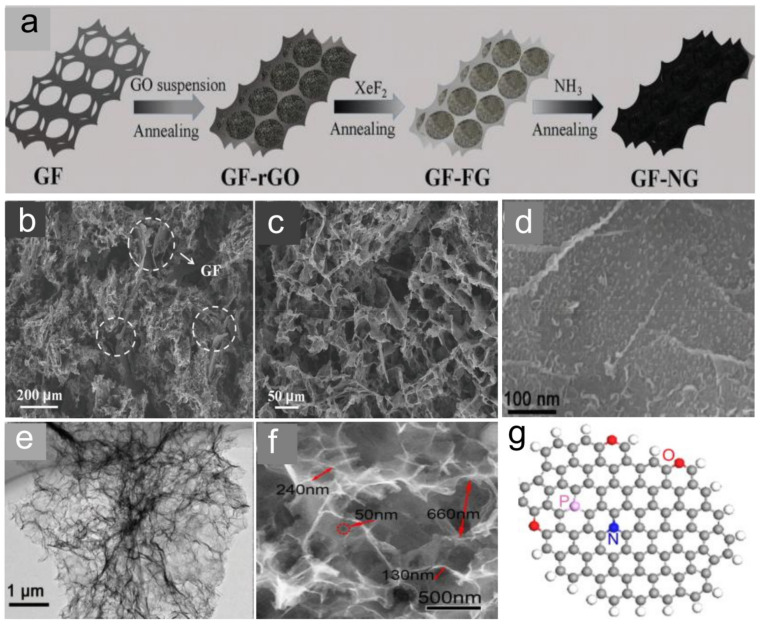
(**a**) Schematic drawing of the procedure for fabricating 3D GF-NG network macrostructure. (**b**) Low-magnification and (**c**) high-magnification cross-sectional FESEM images of GF-NG. The sections marked by dots circles are the skeletons of GF [251] (Copyright 2017 WILEY-VCH Verlag GmbH & Co. KGaA, Weinheim). SEM (**d**) and TEM (**e**) images of N-doped graphene (Copyright 2017 WILEY-VCH Verlag GmbH & Co. KGaA, Weinheim) [259]. (**f**) SEM image of 3D hierarchical porous graphene. (**g**) Lowest Unoccupied Molecular Orbital of N-P-O co-doped graphene [260] (Copyright 2016 Elsevier Ltd., All rights reserved).

Nitrogen doping is considered as an effective strategy to enhance the electrochemical performance of graphene. Doping not only affects the electronic structure and properties of graphene, but it also reduces the degree of aggregation and results in a morphology that allows easy access to electrolyte ions [257]. Some researchers have demonstrated that N-doping could greatly improve the specific capacitance of graphene. In 2013, Lu et al. [258] developed a solvothermal method to prepare N-doped graphene and achieved a specific capacitance of 301 F g^−1^. In 2015, Qin et al. [241] reported a thermal treatment method to prepare robust 3D N-doped graphene (R-3DNG) and achieved a specific capacitance of 509 F g^−1^, which is approaching the theoretical capacitance of graphene (550 F g^−1^). In 2016, Wang et al. [242] prepared N-doped graphene by treating polypyrrole-modified GO with plasmas, and observed a specific capacitance of 312 F g^−1^. In 2017, Hang et al. [251] reported an N-superdoped 3D graphene network structure (3D GF-NG) by immersing highly conductive GF with a 3D interconnected network structure into an aqueous solution of GO sheets (Figure 6a). The N-doped GO aerogels closely connected with the GF skeleton, forming an interconnected GF-NG network structure (Figure 6b,c), and demonstrating a large specific surface area of 583 m^2^ g^−1^, through which electrolyte ions could easily access to the surface of graphene to form electric double layers [251]. The fabricated 3D N-doped rGO electrode delivered a high specific capacitance of 312 F g^−1^ at 5 mV s^−1^ [251]. In 2018, Zhang et al. [259] reported an advanced N-doped graphene electrode with an ultrahigh specific capacitance of 481 F g^−1^ at 1 A g^−1^, and with superior cycling stability of 98.9% capacitance retention after 8000 cycles. Figure 6d shows the SEM image of the N-doped graphene nanosheets, revealing many densely stacked gibbous bubbles with one dimension of about 10 nm on the surface of undulated graphene nanosheets [259]. The TEM image of the N-doped graphene in Figure 6e illustrates its unique wrinkled structure. This unique structure improves the access of ions between the electrolyte and electrode surface and thereby enhances the transport rates toward the interface of the electrode [259]. The as-produced N-P-O co-doped 3D hierarchical porous graphene by Zhao et al. [260] exhibited many nanopores (Figure 6f), facilitating the high volumetric density of the product. As shown in Figure 6g, the density functional theory (DFT) calculations were performed to investigate the co-doping effect of N-P-O in the pristine graphene. All atoms in the models were free to fully relax [260]. The unique 3D hierarchical porous graphene electrode delivered an ultrahigh specific capacitance of 426 F g^−1^. 

Modifying the surface structure of electrode materials could improve their compatibility with electrolytes, enrich redox sites, and enhance the surface conductivity, leading to good electrochemical performance [33]. Recently, oxygen- and nitrogen-containing groups have been well studied to modify the graphene surface. For example, Song et al. [254] recently reported different functionalized graphene networks by using amine molecules and a facile two-step hydrothermal method. The as-fabricated graphene composite exhibited an improved capacitance and fast ionic diffusion features in aqueous and organic electrolytes, with less than 10% capacitance decay during 10,000 charge/discharge cycles [254]. Li et al. [257] reported chemical compounds of GO and amine molecules as spacers by one-step hydrothermal reactions. The as-prepared graphene composite electrode exhibited excellent performance with a high specific capacitance of 597 F g^−1^ [255]. In conclusion, heteroatoms in doped graphene materials play a key role in electron transfer and energy conversion processes. The incorporation of nitrogen or molecular modifiers can provide the work electrodes with high-density active sites to enhance the capacitance performance. Moreover, it can also reduce the agglomeration level of graphene and create few-layer graphene sheets with interconnected open pores, which provide an effective pathway for charge transport.

## 3. Design Structures of Hybrid Supercapacitors

**Figure 7:**
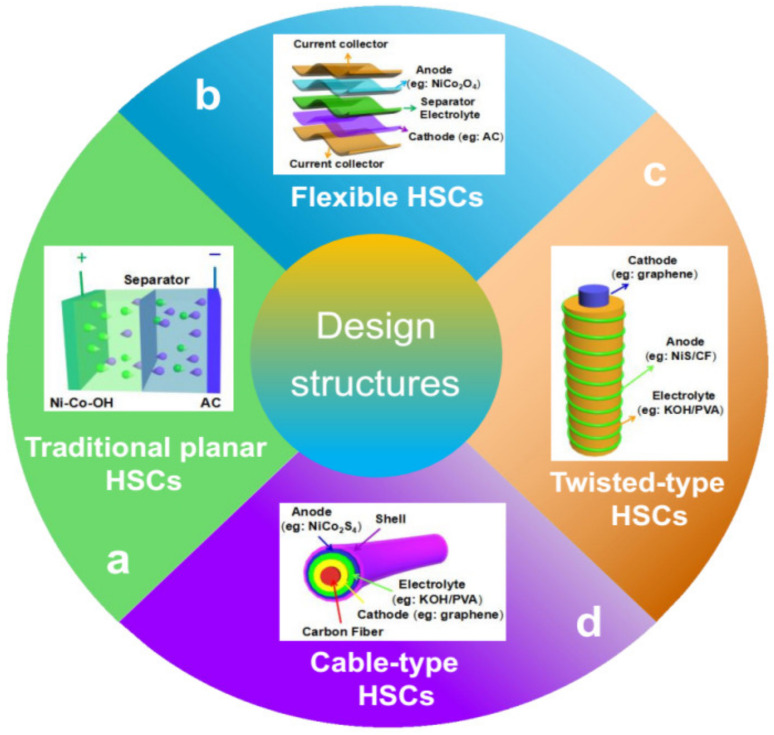
The hybrid supercapacitors with four representative structure types, namely, (**a**) traditional planar HSCs, (**b**) flexible HSCs, (**c**) twisted-type HSCs, and (**d**) cable-type HSCs.

A HSC device usually contains positive and negative electrodes, an electrolyte, a separator (to prevent short circuits between electrodes), and current collectors. Besides the electrodes, electrolytes also play an important role in HSC performance. The electrolytes of HSCs could be organic (LiPF_6_, LiBF_4_, LiClO_4_, NaClO_4_, NaPF_6_, etc.), ionic liquid (BMIMBF_4_), gel-polymer (PVA-H_3_PO_4_, PVA-LiCl, etc.), or aqueous of acidic (H_2_SO_4_, CH_3_SO_3_H), alkaline (KOH, NaOH), and neutral (Na_2_SO_4_, Li_2_SO_4_) [13]. Aqueous electrolytes usually have the advantages of high ionic conductivity, low cost, non-flammability, safety, and convenient assembly in air [261]. But its potential window is limited to 1.2 V, which is far lower than that of organic electrolytes (3.5–4 V). A high-potential window is a large merit for organic electrolytes, which could significantly contribute to high energy density. However, it is less conductive, expensive, usually flammable, and more toxic [13,261]. Ionic liquids as nonvolatile, highly stable electrolytes are considered as the most promising electrolytes compared to organic ones for HSC applications [13]. The gel-polymer electrolyte is usually used for designing and fabricating flexible/stretchable or even smart HSCs due to its merits of avoiding electrolyte leakage or without using an additional separator [262,263]. In this section, four main types of HSCs (Figure 7) are summarized and discussed in detail.

### 3.1. The Traditional Planar HSC Devices

**Figure 8:**
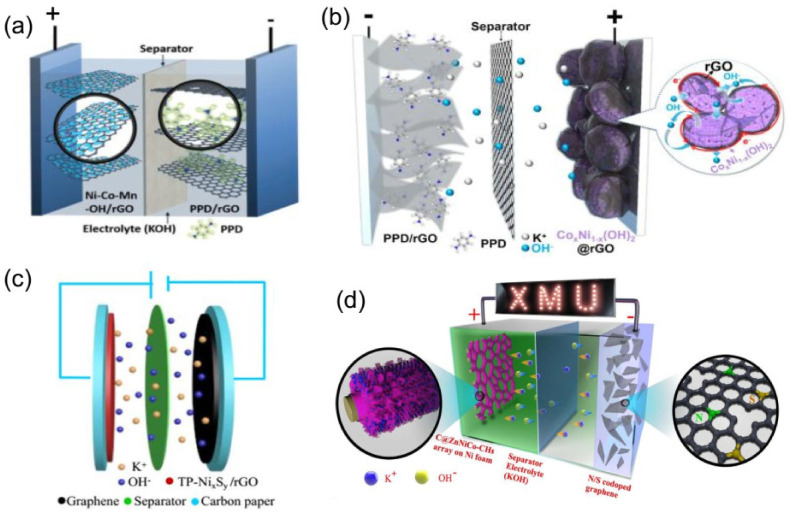
(**a**) Schematic illustration of the HSC device using Ni-Co-Mn-OH/rGO as positive electrode and PPD/rGO as negative electrode [50] (Copyright 2017 WILEY-VCH Verlag GmbH & Co. KGaA, Weinheim). (**b**) Schematic illustration of the hybrid supercapacitor device with a Co_x_Ni_1−x_(OH)_2_@rGO composite as a battery-type faradaic electrode and a p-phenylenediamine (PPD)-modified rGO composite as a capacitive electrode [153] (Copyright 2016 Elsevier B.V.). (**c**) Schematic illustration of the NiS-Ni_3_S_2_-Ni_3_S_4_/rGO//graphene hybrid supercapacitor device [12] (Copyright 2017 Elsevier Ltd., All rights reserved). (**d**) Schematic illustration of the C@ZnNiCo-CHs//N,S-codoped rGOs hybrid supercapacitor device [266] (Copyright 2018, published by Elsevier B.V.).

The traditional HSC devices are constructed from two different types of planar electrodes, one membrane charge separator, and an electrolyte sandwiched together. They have demonstrated great potential in hybrid electric vehicles (electric buses and trains), the aerospace industry, and portable electronic devices [264,265]. To date, remarkable progress has been made in the development of high-performance HSCs. For example, Zhao et al. [50] reported a high-performance HSC device based on Ni-Co-Mn-OH/rGO as the positive electrode and PPD/rGO as the negative electrode (Figure 8a). The rate capability of the HSC device was evaluated by cyclic voltammetry at different scan rates. The shapes of CV curves were well maintained with the increase in scan rate from 5 to 100 mV s^−1^, revealing the high-rate capability of the HSC device. Moreover, the as-fabricated device exhibited an energy density of 74.7 W h kg^−1^ at a power density of 1.68 kW kg^−1^, while maintaining a capacity retention of 91% after 10,000 cycles at a charge-discharge current density of 20 A g^−1^. In addition, they also fabricated a high-performance HSC device based on Co_x_Ni_1−x_(OH)_2_@rGO composite as a battery-type faradaic electrode and a p-p henylenediamine (PPD)-modified rGO composite as a capacitive electrode (Figure 8b) [153]. The shapes of CV curves were well reserved as the scan rate was increased from 5 to 100 mV s^−1^, indicating reasonably high-rate capability of the hybrid supercapacitor, as a result of the electrochemical properties of both the positive and negative electrodes [153]. Moreover, the as-fabricated HSC device also demonstrated a high energy density of 72 Wh kg^−2^ and excellent cycling life [153]. Recently, we also designed a HSC device, constructing it from NiS-Ni_3_S2-Ni_3_S_4_/rGO (Ni_x_S_y_/rGO) as a battery-type faradaic electrode and graphene as a capacitive electrode (Figure 8c), which exhibited a similar electrochemical behavior in a voltage range of 0–1.6 V, a high energy density of 46 Wh kg^−1^ at a power density of 1.8 kW kg^−1^, and good cycling stability [12]. Furthermore, we also fabricated a novel HSC device based on C@ZnNiCo-CHs as the positive electrode and N,S-codoped rGOs as the negative electrode (Figure 8d), which delivered an excellent electrochemical behavior in a voltage range of 0–1.6 V, a high energy density of 70.9 Wh kg^−1^, and excellent cycling stability [266]. These findings not only provide a promising electrode material for high-performance HSCs, but also open a new avenue toward knowledge-based design of efficient electrode materials for other energy storage applications [50]. In brief, the traditional planar HSCs are beneficial for achieving a high ratio of energy delivery at high charge-discharge rates, but they are usually large in size, heavy in weight, and mechanically inflexible [7]. Therefore, an important goal for HSC is to develop the small-sized, portable, and flexible devices.

### 3.2. The Flexible HSC Devices

**Figure 9:**
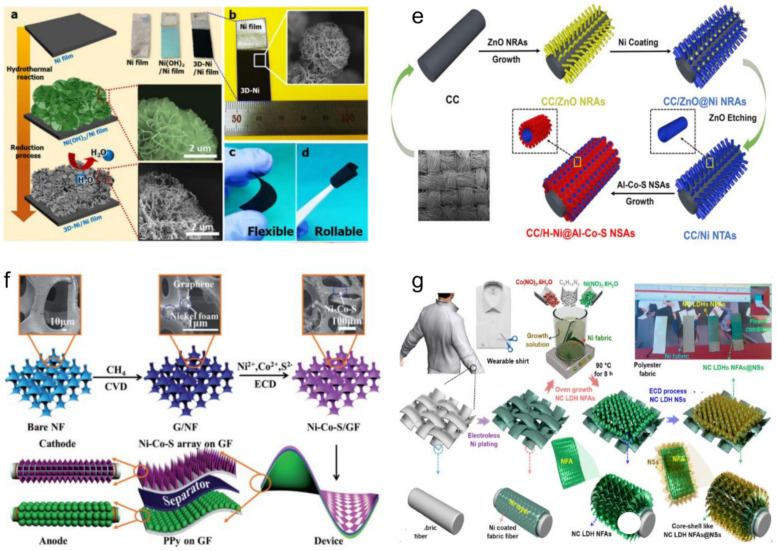
The fabrication and morphology of the mesoporous 3D-Ni current collector. (**a**) Schematic illustration of the fabrication procedure of the mesoporous 3D-Ni/Ni film (inset images are photographs and SEM images of the Ni(OH)_2_/Ni film and the 3D-Ni/Ni film). (**b**) Photograph of the synthesized 3D-Ni/Ni film (the inset is a magnified SEM image). (**c**) Photograph of the 3D Ni/Ni film with flexible (**d**) and rollable properties [270] (Copyright 2017 Elsevier Ltd., All rights reserved). (**e**) Schematic illustration of the synthesis of the petal-like Ni-Co-S and the construction of HSC devices [271] (Copyright 2017 WILEY-VCH Verlag GmbH & Co. KGaA, Weinheim). (**f**) Schematic illustration of the fabrication of a hierarchical core-branch CC/H-Ni@Al-Co-S nanosheet electrode [272] (Copyright 2018 American Chemical Society). (**g**) Schematic illustration of the fabrication process of NC LDH NFAs@NSs/Ni fabric using a wearable polyester shirt [275] (Copyright 2017 American Chemical Society).

Owing to the rapid growth of portable and wearable consumer electronics, such as wearable displays, on-body sensors, artificial electronic skin, and distributed sensors, enormous effort has been devoted to flexible, wearable, and integratable electronics to meet the demands of modern society [267,268,269]. In recent years, some research has been done to fabricate stretchable HSC devices, mostly by using carbon fibers (CFs), nickel foam, or conductive polymers as substrates to achieve stretchability [270,271,272,273,274]. For example, Kim et al. [270] recently developed a flexible electrode based on binder-free nickel cobalt layered double hydroxide nanosheets adhered to nickel cobalt layered double hydroxide nanoflake arrays on nickel fabric (NC LDH NFAs@NSs/Ni fabric) using facile and eco-friendly synthesis methods (Figure 9a–d). The fabricated HSC device, constructed from the NC LDH NFAs@NSs/Ni fabric positive electrode and MnO_2_/3D-Ni negative electrode, exhibited excellent electrochemical durability and flexibility. Zhang et al. [271] fabricated the HSC device based on the Ni-Co-S/graphene foam (GF) as the positive electrode and polypyrrple (PPy)/GF as the negative electrode (Figure 9e), which demonstrated robust flexibility under different bending angles. Recently, carbon-based fibers have also been widely used in flexible energy storage electrodes due to their unique features involving the high flexibility, good mechanical properties, and its unchanged sheet resistance even in a very high bending state [9]. For example, Huang et al. [272] designed and fabricated hierarchical core-branch Al-doped cobalt sulfide nanosheets anchored on Ni nanotube arrays combined with carbon cloth (denoted as CC/H-Ni@Al-Co-S) (Figure 9f), which exhibited high specific capacity (1217 C g^−1^ at 1 A g^−1^). Moreover, the as-fabricated CC/H-Ni@Al-Co-S//graphene/CNT HSC device not only demonstrated high flexibility, but also delivered a high energy density of 65.7 Wh kg^−1^. Nagaraju et al. [275] reported the progress toward a high-performance HSC device based on a 3D Ni-electrode (Figure 9g), which delivered an excellent energy density of 75 Wh kg^−1^ and a high-power density of 5.3 kW kg^−1^. Furthermore, the device also demonstrated excellent flexibility and a potential application for wearable energy management.

Up to date, various studies have been carried out on flexible planar HSC devices, but the cycling stability of HSCs still needs to be improved. The phase transformation, structural collapse, and volumetric expansion may be the most key factors that causing the reduction of capacity during long-term charging-discharging cycles [273]. The incorporation of metal cations into hybrid electrode materials can effectively prevent the phase transitions in active materials, which can improve their long-term cyclability [275]. Another effective method is to fabricate the nanostructured composites with graphene [275], which can effectively prevent the nanostructure from collapsing and avoid the corrosion of energy storage capacity.

### 3.3. The Twisted-Type HSC Devices

**Figure 10:**
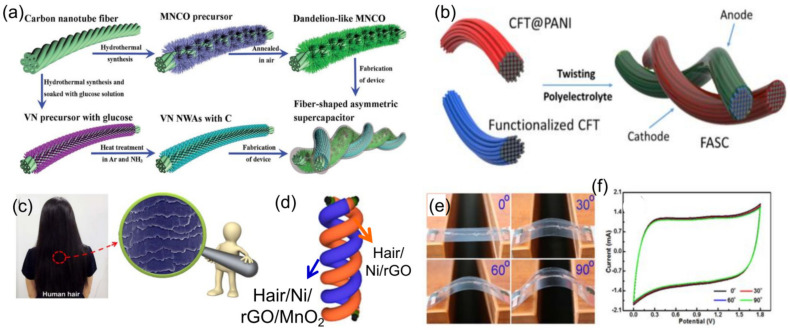
(**a**) Detailed schematic illustration of the fabrication process for the twisted-type HSC device [280] (Copyright 2017: The Royal Society of Chemistry). (**b**) Schematic illustration of the fabrication of the twisted-type HSC device [281] (Copyright 2015 Elsevier Ltd. All rights reserved). (**c**) Photograph of human hair, and (**d**) schematic diagram of the HSC devices constructed by twisting the human hair/Ni/rGO/MnO_2_ fiber and human hair/Ni/rGO fiber together [282] (Copyright 2017 Elsevier Ltd. All rights reserved). (**e**) Schematic illustration of the fabrication of twisted-type HSC device. (**f**) CV curves (at 15 mV s^−1^) under different bending conditions [283] (Copyright 2016 The Royal Society of Chemistry).

Unlike conventional rigid planar HSCs, the twisted-type HSCs can be directly used as flexible power sources in wearable, self-powered electronic devices [275]. They can be either co-woven/knitted into existing fabrics/textiles or they can be woven/knitted by themselves [276]. Recently, many advanced fiber-shaped HSC devices have been widely reported in the literature [276,277,278,279,280]. For instance, Sun et al. [280] reported a twisted-type HSC device assembled by twisting a molybdenum-nickel-cobalt ternary oxide/carbon nanotube fiber (MNCO/CNTF) positive electrode and thin carbon-coated vanadium nitride (VN) nanowire arrays on a CNTF negative electrode (Figure 10a), which delivered a high specific capacitance of 490.7 F cm^−3^ (1840 mF cm^−2^) at a current density of 1 mA cm^−2^ and outstanding flexibility and stability with capacitance retention maintained at 90.2% after bending 3500 times. Jin et al. [281] reported a twisted-type HSC with PANI-coated carbon fiber thread as the positive electrode and functionalized carbon fiber thread as the negative electrode (Figure 10b), which also demonstrated high flexibility and stability. Liu et al. [282] recently reported a novel flexible twisted-type HSC with coaxial human hair/Ni/Graphene/MnO_2_ fiber as the positive electrode and coaxial human hair/Ni/Graphene fiber as the negative electrode, which first reveals that human hair could also be used to fabricate flexible HSCs. The as-fabricated HSC exhibited excellent rate capability (up to 20 V s^−1^), high volumetric energy density (1.81 mWh cm^−3^), and excellent flexibility [282]. Senthilkumar et al. [283] fabricated the twisted-type HSC assembled by copper hexacyanoferrate coated carbon fibers (CuHCF@CFs) and porous carbon coated carbon fibers (PC@CFs) electrodes (Figure 10e), which also demonstrated outstanding flexibility (Figure 10f) and a high energy density of 10.6 Wh kg^−1^. In general, the flexible twisted-type HSCs are attractive as power sources for miniaturized electronic devices, because they have small volumes and could be easily integrated into variously shaped structures [284]. However, these HSCs still suffer from relatively low capacitance and a high production cost, which cannot meet the ever-increasing demand for flexible devices. In addition, some technical challenges still limit the development of strong, flexible, and wearable HSC devices with high performance. Therefore, more efforts are needed in searching for new structured active materials with high electron conductivity, high electrochemical sites, and novel fiber current collectors with strong mechanical stability and ultra-high flexibility to develop fiber-shaped flexible HSC devices with high performances.

### 3.4. The Cable-Type HSC Devices

**Figure 11:**
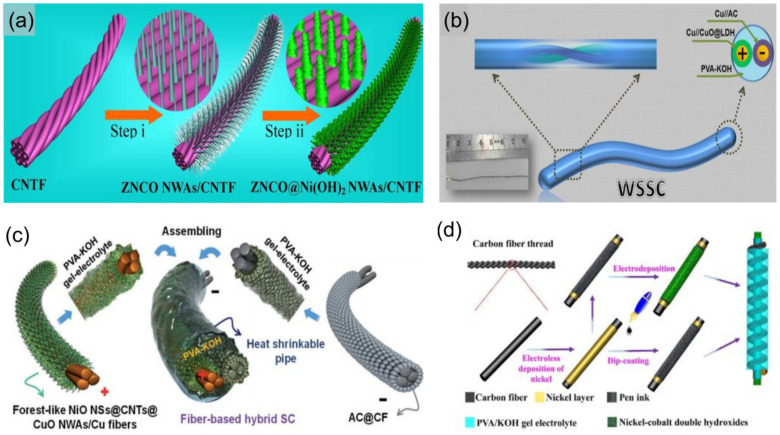
(**a**) Schematic fabrication process of the ZNCO@Ni(OH)_2_ NWAs on a carbon nanotube fiber [287] (Copyright 2017 American Chemical Society). (**b**) Schematic representation of the flexible cable-type HSC device based on CuO@CoFeLDH and active carbon electrodes [288] (Copyright 2016 Elsevier Ltd., all rights reserved). (**c**) Schematic diagram showing the fabrication process of the HSC device [289] (Copyright 2018 WILEY-VCH Verlag GmbH & Co. KGaA, Weinheim). (**d**) Schematic diagram of the fabrication procedure of the HSC device [290] (Copyright 2017 American Chemical Society).

Among various flexible energy storage devices, the cable-type HSCs have attracted increasing attention due to their merits of low weight, tiny volume, high flexibility, and wearability [285,286]. So far, many advanced cable-type SC devices have been widely reported in the literature [287,288,289,290]. For instance, Zhang et al. [287] recently reported a facile and cost-effective method to directly grow three-dimensionally well-aligned zinc-nickel-cobalt oxide (ZNCO)@Ni(OH)_2_ nanowire arrays on a carbon nanotube fiber (Figure 11a) with an ultrahigh specific capacitance of 2847.5 F cm^−3^ (10.678 F cm^−2^) at a current density of 1 mA cm^−2^. Moreover, they also fabricated a novel cable-type HSC device based on ZNCO@Ni(OH)_2_ NWAs/CNTF as the positive electrode and a thin layer of carbon-coated vanadium nitride nanowire arrays on a carbon nanotube strip as the negative electrode, which demonstrated excellent flexibility and stability. Li et al. [288] developed a novel flexible cable-type HSC device based on Cu//CuO@LDH as the positive electrode and Cu//AC as the negative electrode (Figure 11b), which also presented great flexibility and excellent cycling stability. Nagaraju et al. [289] recently also fabricated a cable-type HSC device based on nickel oxide nanosheet grafted carbon nanotube coupled copper oxide nanowire arrays (NiO NSs@CNTs@CuO NWAs/Cu fibers) as the positive electrode and AC as the negative electrode (Figure 11c), which demonstrated excellent flexibility and stability. Gao et al. [290] reported a flexible cable-type HSC (Figure 11d) based on the Ni-Co DHs and pen ink electrodes on metallized CF. Moreover, the as-fabricated device also delivered good cycling stability and high energy [290]. This low-cost and high-performance flexible cable-type HSC provides an alternative strategy toward efficient flexible energy storage devices and wearable energy equipment.

## 4. The Charge-Storage Mechanism of Hybrid Supercapacitors

**Figure 12:**
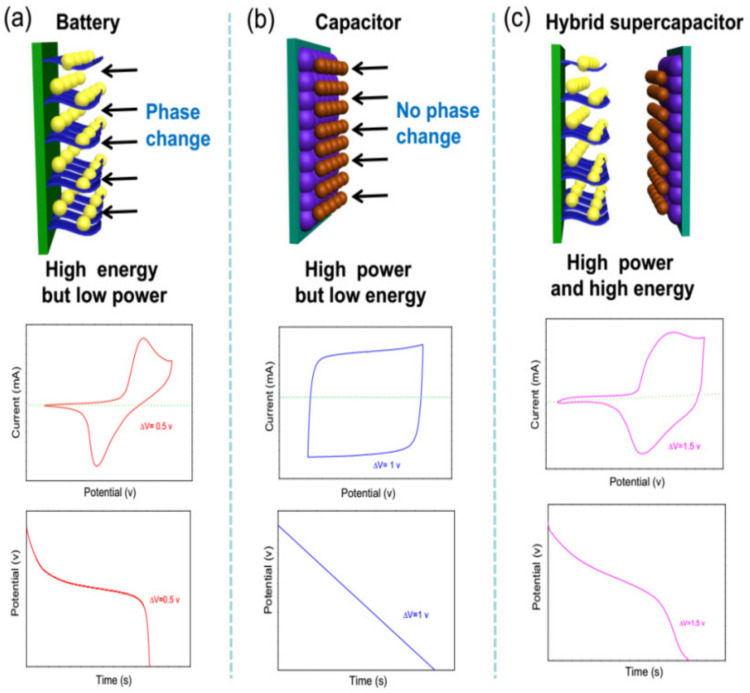
Schematic of charge storage mechanisms, cyclic voltammograms, and corresponding galvanostatic discharge curves for batteries (**a**), capacitors (**b**), hybrid supercapacitors (**c**).

For a better understanding of the operative mechanisms of the combination of Faradaic and capacitive electrodes in hybrid supercapacitors, some basic theoretical aspects will be discussed in this section. Moreover, how to distinguish between a capacitor-like and a battery-like electrode materials will also be presented in the next paragraphs. Figure 12 depicts the characteristic behavior of these conventional energy storage materials. The capacity of the batteries relies predominantly on the Faradaic reaction that is made possible by the intercalation/de-intercalation of charge-compensating ions (H^+^, Li^+^, or Na^+^) within the crystalline structure of electrode materials [27]. However, the capacitance of the supercapacitors mainly depends on the electrochemical adsorption/desorption of cations and anions at the electrode/electrolyte interface (double-layer capacitive) or surface faradaic redox reactions (pseudocapacitive) at the surface of electrode materials [7,27]. Some researchers may ask how to determine whether a now material that it should be classified as a battery-type or a capacitor-type material. As we all know, the electrochemical behaviors of the batteries and supercapacitors are both characterized by the cyclic voltammetry (CV) and galvanostatic charge/discharge (GCD) tests. A related analysis of the energy storage mechanisms can be performed from the CV curves. The kinetic information obtained from the peak current (*i*) response can be summarized using the following equation [4,27]:

(1) $$i=av^{b}$$

where the measured current *i* at a fixed potential obeys a power law relationship with the potential sweep rate *v* [27]. The *b*-value is determined by the slope of the log(*v*)–log(*i*) plots. In general, the *b* value of 0.5 represents a semi-infinite diffusion behavior, whereas 1.0 indicates a capacitive process [50,267]. The peak current (*i*) response of battery-type materials exhibits classic semi-infinite diffusion because of phase transformations, thus resulting in low Coulombic efficiency and poor rate performance. The CV curves of the corresponding electrodes show prominent and widely separated peaks associated with the reduction and oxidation, and the discharge curves exhibit obvious plateaus (Figure 12a) [7]. However, the current (*i*) response of capacitor-type materials is not controlled by the diffusion process, and it exhibits a linear current response dependency on the scan rate [7]. The CV curves of the corresponding electrodes show a rectangular or approximate rectangular shape, and the discharge curves exhibit a linear voltage response during constant current charge-discharge (Figure 12b). Hence, the capacitor-type electrode materials exhibit high power density but poor energy density, whereas the battery-type materials show high energy density but poor power density.

As a patent for an energy-storage device that combined a double-layer capacitor electrode with a positive nickel battery was reported by Varakin et al. in the mid-1990s [291]. In during the past few years, similar energy-storage devices have been widely reported in numerous publications. Unfortunately, the concept of such energy storage devices is very confusing in many publications. As suggested by Brousse et al. [7], the term of hybrid supercapacitors should be used when pairing two electrodes with various charge storage behaviors (i.e., one faradaic and one capacitive). The concept of an asymmetric supercapacitor covers a wider range of electrode combinations because it can be used for supercapacitors using two electrodes of the same nature but with various mass loadings, or two electrodes using various materials [7]. It should be emphasized that a relatively complex charge-storage mechanism occurs in the hybrid supercapacitor devices and some hybrid nanocomposite electrodes, and the corresponding electrochemical characteristics are neither purely capacitor-type nor purely battery-type. As a new type of hybrid charge storage mechanism, the current (*i*) response to the sweep rate (*v*) will depend on the electrochemical reaction associated with diffusion-controlled and surface-controlled (capacitive) reaction. Therefore, the current response (*i*) at a fixed sweep rate (*v*) can be summarized according to the following equation [50,267]:

(2) $$i(v)=k_{1}v^{1/2}+k_{2}v$$

By determining both *k*_1_ and *k*_2_, we can distinguish the fraction of the current arising from cation intercalation ($k_{1}v^{1/2}$) and that from capacitive ($k_{2}v$) processes at each potential [267]. The total energy stored in the hybrid supercapacitors is the sum of the energy stored in the battery-type electrode and that of the capacitor-type electrode (Figure 12c). The battery-type electrode is used to improve the energy densities compared to those of typical double-layer capacitors and pseudocapacitors. On the other hand, the capacitor-type electrode is used to improve the power densities of the cells compared to the typical batteries. The main reason is that the capacitor component can improve the electron transfer to the battery component in the hybrid system, causing a better charge transfer reaction at high rates. Hence, the hybrid supercapacitors can usually exhibit high power densities.

Obviously, the total energy storage capacity and rate capability of the hybrid supercapacitors can be optimized by expanding the operating potential window and designing porous hybrid nanostructured composite materials. The operating potential window of the hybrid supercapacitors can be extended to ~1.5 V or even higher (Figure 12). The hybrid nanostructured electrodes, which combine battery components (transition metal oxides/sulfides) with capacitor components (carbon-based), usually exhibit higher electrochemical performance, especially high-rate performance and cycle life. The charge storage of this hybrid electrode will be due to both battery and capacitor components: firstly, the capacitor component is charged via electrostatic forces until the electrode potential reaches the redox reaction potential of the battery component [292]. Then, the battery component is charged through the Faradiac reaction until the faradaic component reaches its full-charge state [293]. It should be emphasized that calculating the capacitance of such electrodes by using the equation derived for capacitors will lead to greatly overestimated capacitance values that can be found in many publications. As suggested by Brousse et al. [7], the specific capacity (C) instead of the capacitance of such electrodes should be calculated according to the following equations:

(3) $$Q=i_{m}\Delta t$$

where *i_m_* = *I/m* (A g^−1^) is the current density, *m* is the mass of the active electrode material, *I* is the current, and Δ*t* is the discharge time [12,50]. Regarding the detailed information, Brousse et al. have given an excellent comment in their publication [7].

With the development of renewable energy and electrified transportation, it can be expected that the energy conversion and energy storage devices will become more and more important in our daily lives. In the future, energy storage systems will mainly focus on hybrid devices combining the best features of battery-type Faradaic electrodes and capacitive electrodes. Understanding of the synergistic effect among different active components on the electron transportation and surface reactions is very challenging and significant. For the negative electrode, the challenge is still increasing the capacitance, which is critical for charge/weight/volume balance with the positive electrode to maximize the energy density of the device. Non-planar hybrid electrode architectures will play an important role in future energy storage systems. Conventional electrodes cannot satisfy the development of flexible and lightweight devices in modern electronics; it is still a challenge to develop a highly flexible and portable integrated energy package. The integration of HSCs with other multifunctions such as electrochromism, shape memory, photo self-charging, thermal self-protective, and self-healing will be significant and require further study.

## 5. Conclusions

In this review, we summarized the development of recent advances in HSCs, including the electrode materials, such as transition metal oxides/sulfides/hydroxides and carbon-based materials (activated carbon and graphene), the working principles/mechanisms, and purposeful design/optimization. In general, the HSCs have been developed as attractive high-energy storage devices combining a typical battery-type electrode with a large positive cutoff potential and a capacitive electrode with a high overpotential in the negative potential range, rendering a significant increase in the overall cell operating voltage.

The traditional planar architectures are very limited in the thickness of the Faradaic electrode due to the low electrical and ionic conductivities, which retard the overall device kinetics. Nanostructuring, especially 3D hybrid architectures, reducing the mass-transfer resistances and ion diffusion pathways, and increasing the electrical conductivity and stability, are good strategies to solve this issue. In addition, it can also can promote rapid electron and ion accessibility to electrochemically active sites and ease the electron hopping between neighboring nanoparticles. Nanostructured composite materials are considered as the most promising candidates for fabricating high-performance HSC devices. However, complex preparation methods and fabrication processes hinder their wider practical application. The challenge is to explore novel electrode materials or architectures to fabricate high-performance HSCs using a cost-effective technology.

Recently, enormous efforts have been devoted to the significant advancement in flexible electrode design and device construction for high-performance HSCs. The development of flexible, portable, and wearable energy storage devices has paved the way for the further applications of renewable energy to power electric cars and enable the internet of things. However, there are still some challenging issues for promoting the practical commercial application of HSCs. For example, the morphology and structure of nanomaterials are sometimes difficult to maintain during the long cycles because of their poor mechanical and chemical properties. In addition, the structural evolution and degradation information of electrode materials is difficult to uncover during the long cycling process, and advanced tools are to be developed to reveal the mechanisms of some complex phenomena. Therefore, future efforts should be focused on providing comprehensive insight into the fundamental understanding of the relationship between the electrochemical properties and the structure.

## Figures and Tables

**Table 1:** Specific capacity of Ni(OH)_2_ electrodes.

Electrode Materials	Electrolyte	Voltage (V)	Current Load or Scan Rate	Specific Capacity (C g^−1^)	Reference
3D nanoporous Ni(OH)_2_	6.0 M KOH	0–0.5	7 A g^−1^	759.5	[55]
Ni(OH)_2_ nanospheres	1.0 M KOH	0–0.5	20 A g^−1^	934	[56]
α-Ni(OH)_2_ nanobristles	1.0 M KOH	0–0.45	2 A g^−1^	940.5	[57]
Ni(OH)_2_ microspheres	2.0 M KOH	0–0.55	0.5 A g^−1^	704.5	[58]
Mesoporous a-Ni(OH)_2_	2.0 M KOH	0–0.55	0.5 A g^−1^	983.9	[59]
Ni(OH)_2_ nanoboxes	2.0 M KOH	0–0.5	1 A g^−1^	1247.5	[60]
α-Ni(OH)_2_ nanowires	2.0 M KOH	0–0.4	1 A g^−1^	889.2	[61]
Ni(OH)_2_ nanosheets	6.0 M KOH	0–0.5	2 A g^−1^	825.6	[62]
Ni(OH)_2_ nanoflakes	1.0 M KOH	0–0.4	1 A g^−1^	566.4	[63]
Ni(OH)_2_ nanocubes	3.0 M KOH	0–0.45	1 A g^−1^	828.9	[64]
Amorphous α-Ni(OH)_2_	2.0 M KOH	0–0.35	2 A g^−1^	818.3	[65]
Cabbage-like α-Ni(OH)_2_	1.0 M KOH	0.2–0.6	1 mA cm^−2^	761.2	[66]
Ni(OH)_2_ nanosheets	2.0 M KOH	0–0.45	1 A g^−1^	1072.9	[67]
Ni(OH)_2_ platelets	2.0 M KOH	0–0.6	0.5 A g^−1^	1160.4	[68]
β-Ni(OH)_2_ nanosheets	6.0 M KOH	0–0.6	5 mV s^−1^	1041	[69]
α-Ni(OH)_2_	2.0 M KOH	0–0.5	2 mV s^−1^	267	[70]
α-Ni(OH)_2_ microspheres	6.0 M KOH	0–0.4	1 A g^−1^	992.3	[71]
β-Ni(OH)_2_ nanosheets	6.0 M KOH	0–0.4	5 mA cm^−2^	790.3	[72]
β-Ni(OH)_2_	6.0 M KOH	−0.05–0.35	1 A g^−1^	712	[73]

**Table 2:** Summary of performances of Ni(OH)_2_/rGO composites.

Electrode Materials	∆E (V)	Maximum Capacity (C g^−1^)	Capacity Retention	Cycle Stability	Ref.
Ni(OH)_2_ nanoplatelets/rGO	0.45	955 C g^−1^ (1 A g^−1^)	58.6% (80 A g^−1^)	102% (5000 cycles)	[74]
3D Ni(OH)_2_/rGO network	0.5	563 C g^−1^ (0.5 A g^−1^)	61.8% (10 A g^−1^)	87% (1000 cycles)	[75]
Ni(OH)_2_/rGO	0.6	941 C g^−1^ (4 A g^−1^)	27% (11.2 A g^−1^)	75% (1000 cycles)	[76]
Ni(OH)_2_/rGO aerogel	0.5	516 C g^−1^ (0.5 A g^−1^)	54.3% (2 A g^−1^)	95% (2000 cycles)	[77]
Ni(OH)_2_/3D rGO	0.5	690 C g^−1^ (1 A g^−1^)	86.7% (60 A g^−1^)	78% (1000 cycles)	[78]
Ni(OH)_2_ nanoparticles/rGO	0.38	858 C g^−1^ (0.5 A g^−1^)	52.7% (10 A g^−1^)	89% (1000 cycles)	[79]
Ni(OH)_2_ nanosheets/rGO	0.45	838 C g^−1^ (0.8 A g^−1^)	62.3% (6.4 A g^−1^)	92% (2000 cycles)	[80]
Ni(OH)_2_ nanocrystals/rGO	0.5	951.5 C g^−1^ (1 A g^−1^)	60.9% (20 A g^−1^)	70% (1000 cycles)	[81]
Ni(OH)_2_ nanoplates/rGO	0.5	667 C g^−1^ (2.8 A g^−1^)	71% (45.7 A g^−1^)	100% (2000 cycles)	[82]
Ni(OH)_2_/rGO	0.55	1206 C g^−1^ (2 mV s^−1^)	41% (20 mV s^−1^)	95% (2000 cycles)	[83]
Ni(OH)_2_/rGO	0.55	954 C g^−1^ (1 mV s-1)	30% (50 mV s^−1^)	88% (1000 cycles)	[84]
β-Ni(OH)_2_/rGO	0.5	971 C g^−1^ (1 A g-1)	67.9% (40 A g^−1^)	81% (2000 cycles)	[85]
Ni(OH)_2_ nanoflowers/rGO	0.55	598 C g^−1^ (1 A g-1)	58% (10 A g^−1^)	95% (1000 cycles)	[86]
Flower-like Ni(OH)_2_/rGO	0.4	642 C g^−1^ (1 A g-1)	18.7% (30 A g^−1^)	86% (2200 cycles)	[87]
Ni(OH)_2_/rGO	0.45	546 C g^−1^ (5 mV s-1)	35% (100 mV s^−1^)	88% (1000 cycles)	[88]

**Table 3:** Summary of performances of multi-metal LDH nanomaterials and their hybrids.

Electrode Materials	∆E (V)	Maximum Capacity (C g^−1^)	Capacity Retention	Cycle Stability	Ref.
NiCo-LDH/CC	0.5	908 C g^−1^ (1 A g^−1^)	60% (100 A g^−1^)	88% (10000 cycles)	[177]
NiCo-LDH/CFC	0.45	1009 C g^−1^ (1 A g^−1^)	61% (60 A g^−1^)	95% (2000 cycles)	[178]
NiCoAl-LDH	0.6	1237 C g^−1^ (1 A g^−1^)	48.7% (10 A g^−1^)	93% (3000 cycles)	[179]
NiCoAl-LDH@BG-NF	0.5	999 C g^−1^ (6 A g^−1^)	75.3% (20 A g^−1^)	91% (2000 cycles)	[180]
rGO/Ni_0.75−x_Co_x_Al_0.25_-LDH	0.5	772 C g^−1^ (1 A g^−1^)	70% (40 A g^−1^)	89% (2000 cycles)	[181]
MnCo-LDH@Ni(OH)_2_	0.4	928 C g^−1^ (1 A g^−1^)	56.3% (30 A g^−1^)	91% (5000 cycles)	[182]
Ni_0.76_Co_0.24_-LDH	0.5	1279 C g^−1^ (1 A g^−1^)	87.2% (30 A g^−1^)	70% (20000 cycles)	[183]
HCNs@NiCo-LDH	0.5	1095 C g^−1^ (1 A g^−1^)	74.9% (20 A g^−1^)	—	[184]
NiCo-LDH/rGO	0.45	802 C g^−1^ (1 A g^−1^)	76.9% (20 A g^−1^)	74% (1000 cycles)	[185]
NiCo-LDH@CNT/NF	0.4	818 C g^− 1^(1 A g^−1^)	65.2% (20 A g^−1^)	—	[186]
rGO(25)@CoNiAl-LDH	0.45	839.7 C g^−1^ (1 A g^−1^)	72.9% (10 A g^−1^)	100% (5000 cycles)	[187]
LEG/NiCo-LDH	0.45	1099 C g^−1^ (1 A g^−1^)	83.5% (50 A g^−1^)	89% (5000 cycles)	[188]
NixCo_2x_(OH)_6x_@Ni	0.5	1146 C g^−1^ (5 A g^−1^)	68% (100 A g^−1^)	90% (5000 cycles)	[189]
NiCo-LDHs	0.45	780 C g^−1^ (6 A g^−1^)	66.1% (30 A g^−1^)	86% (1000 cycles)	[173]
Ni-Co LDH NSs/CTs	0.5	1052 C g^−1^ (2 A g^−1^)	57% (20 A g^−1^)	90% (2000 cycles)	[190]
NiMn LDH/rGO	0.5	750 C g^−1^ (1 A g^−1^)	45.3% (10 A g^−1^)	90% (5000 cycles)	[191]
CoMn-LDH	0.65	916 C g^−1^ (1 A g^−1^)	71.1% (10 A g^−1^)	93% (3000 cycles)	[192]
10NiAl-LDH	0.4	849 C g^−1^ (0.5 A g^−1^)	70.7% (20 A g^−1^)	92% (10,000 cycles)	[193]
NiCo_2_O_4_@NiCoAl-LDH	0.6	1088 C g^−1^ (1 A g^−1^)	61% (20 A g^−1^)	93% (2000 cycles)	[175]
Ni-Co LDH/graphene	0.6	759 C g^−1^ (1 A g^−1^)	50% (10 A g^−1^)	93% (2000 cycles)	[165]
NiAl-LDH	0.55	1023.8 C g^−1^ (2 A g^−1^)	68.8% (50 A g^−1^)	86% (5000 cycles)	[194]
NiFe-LDH	0.4	1354 C g^−1^ (5 A g^−1^)	53.7% (10 A g^−1^)	43% (500 cycles)	[195]
MnCo-LDH	0.45	230 C g^−1^ (1 A g^−1^)	69.7% (20 A g^−1^)	92% (2000 cycles)	[196]
GO/NiAl-LDHs	0.4	384 C g^−1^ (1 A g^−1^)	67% (10 A g^−1^)	70% (2000 cycles)	[197]
NiAl-LDHs	0.45	277.6 C g^−1^ (1 A g^−1^)	73.6% (20 A g^−1^)	96% (2000 cycles)	[198]
Co-Al-LDH	0.45	377 C g^−1^ (1 A g^−1^)	80.8% (100 A g^−1^)	95% (20,000 cycles)	[199]
A-NiCo-LDHs	0.5	1224 C g^−1^ (1 A g^−1^)	67.1% (20 A g^−1^)	93% (10,000 cycles)	[176]
Ni-Co@Ni-Co LDH	0.5	1207 C g^−1^ (1 A g^−1^)	82.1% (20 A g^−1^)	98% (5000 cycles)	[200]
Ni-Mn LDH/carbon	0.5	634 C g^−1^ (1 A g^−1^)	78.4% (10 A g^−1^)	79% (5000 cycles)	[201]

**Table 4:** Summary of performances of HSCs based on AC as negative electrode.

Device	Voltage (V)	Energy Density (Wh kg^−1^)	Power Density (W kg^−1^)	Cycle Performance	Ref.
Zn-Ni-Al-Co oxide//AC	0–1.5	72.4	533	90% (10,000 cycles)	[207]
NiO/Ni_3_S_2_//AC	0–1.6	52.9	1600	92.9% (5000 cycles)	[208]
Ni(OH)_2_//AC	0–1.3	35.7	490	81% (10,000 cycles)	[57]
Ni(OH)_2_//AC	0–1.6	22	800	85.7% (4000 cycles)	[67]
Ni(OH)_2_-AB//AC	0–1.4	18.7	1971	91% (5000 cycles)	[69]
β-Ni(OH)_2_//AC	0–1.6	36.2	100.6	92% (1000 cycles)	[200]
rGONF/Ni(OH)_2_//AC	0–1.7	44.1	467	77.4% (2000 cycles)	[209]
NiS//AC	0–1.8	31	900	100% (1000 cycles)	[210]
NiS//AC	0–1.6	33.4	800	87.3% (5000 cycles)	[211]
Ni/Co-LDHs//AC	0–1.6	165.5	1530	85% (500 cycles)	[212]
ZnCo_2_O_4_//AC	0–1.6	29.7	398.5	72.5% (1000 cycles)	[213]
ZnCo_2_O_4_//AC	0–1.6	33.98	800	93.3% (10,000 cycles)	[214]
NiCo_2_O_4_/rGO//AC	0–1.5	57	375	90.2% (20,000 cycles)	[137]
NiCo_2_S_4_/Co9S8//AC	0–1.5	33.5	150	65% (5000 cycles)	[144]
CuCo_2_S_4_-HNN//AC	0–1.6	44.1	800	94.1% (6000 cycles)	[215]
MCS/GNF//AC	0–1.6	54.26	1120	81.9% (4000 cycles)	[216]
NiCo_2_S_4_//AC	0–1.6	25.5	334	93.4% (1500 cycles)	[168]
NiCo_2_S_4_//AC	0–1.6	42.7	1583	92% (10,000 cycles)	[170]
NiCo_2_S_4_ nanopetals//AC	0–1.6	35.6	819.5	94.3% (5000 cycles)	[217]
NiCo-LDH//AC	0–1.6	69.7	800	87% (20,000 cycles)	[177]
NiCo-LDH//AC	0–1.5	17.5	10500	91.2% (10,000 cycles)	[167]
MnCo-LDH@Ni(OH)_2_//AC	0–1.5	47.9	750.7	90.9% (5000 cycles)	[182]
NiCo-LDH//AC	0–0.8	15.9	400	82.7% (20,000 cycles)	[183]
NiCo_2_O_4_@NiCoAl-LDH//AC	0–1.6	74.6	800	93% (2000 cycles)	[175]
NiCo-LDH/graphene//AC	0–1.7	68	594.9	94.2% (2500 cycles)	[165]
NiFe-LDH//AC	0–1.6	50.2	800	65% (2000 cycles)	[195]
NiMoO_4_//AC	0–1.7	60.9	850	85.7% (10,000 cycles)	[218]
NiCo-10//AC	0–1.6	51.5	825	89.5% (6000 cycles)	[219]
NiSe_2_//AC	0–1.6	44.8	969.7	87.4% (20,000 cycles)	[220]

## Data Availability

Not applicable.
